# The Teeth, with Respect to the Voice

**Published:** 1847-10

**Authors:** A. Hill


### ARTICLE II.

*The Teeth, with Respect to the Voice.* By A. Hill, D. D. S.

"No man liveth to himself"—is a truth not merely attested by revelation, but confirmed by universal observation and experience. The habits of society—all human governments and laws, together with the relations and responsibilities growing out of them, all combine to establish this truth. But these evidences, are not more conclusive and irrefragable in relation to the above named proposition, than are those which go to prove the close and intimate relation subsisting between the teeth and the other distinct organs of the human body. Groups and congeries of organs, associated together for the accomplishment of certain specific objects, may sustain a relation to the whole, similar in character to that which is represented by *families* and *neighborhoods*. Others, again, to that which subsists between *states* of the same confederacy, and others to the *supreme government* under which they live. The *head*, the *limbs*, and the *trunk*, are all related to each other, and the different organs or parts of each are the instruments of the whole.

We mention not these things, with a view to instruct the physiologist, who so *well* knows the truth of what we state, or with a view to claim originality of thought in this respect; but for the purpose, simply, of refreshing his memory, and to deduce certain conclusions and facts, which we design to spread before the readers of the Journal. And surely nothing can be more interesting to the student of physiology, than to trace the remarkable phenomena, exhibited by this wonderful machine, in its combined and curious relations. And this interest is greatly enhanced, as we are enabled, from time to time, to trace some of those relations, not merely as they exist between different organs in the performance of animal functions alone, but in their higher modes of manifestation, especially, when they become the vehicles of thought, and language, or instruments, through which the soul of man displays itself in a most wonderful manner. The faculty of speech in man, and the intelligence which prompts to the exercise of articulate sounds in communication with his fellow, are his crowning glory. No attribute of humanity is more distinctive—none more remarkable. Around it clusters every thought, and every feeling of the human soul, and it displays, like a faithful microcosm, the intellectual world in miniature. These considerations alone, it seems to us, are sufficient to invest this subject with singular importance, and to justify the effort which we make, to show the relation, which the teeth sustain to *speech* and *elocution*.

The exercise of the vocal organs in man, is so intimately connected with the dental apparatus, and the powerful influence which the latter exerts over the former, especially in the use of articulate sounds, is so great, that it becomes somewhat marvellous that the attention of physiologists, and elocutionists, should have been so long withheld from it. But as dental science advances, we hope the time is not far distant, when its legitimate claims to importance in this, as well as many other respects, will be not only felt and appreciated, but acknowledged as it deserves, and of right ought to be.

That the perfection of speech, and the absolute command of the voice, is a desirable attainment—nay, more—a splendid accomplishment, no one will deny. To feel its magic power, or to be brought within the circle of its charm, we have not to go back to the palmy days of Greece and Rome, and listen with the wrapt throng that clusters, in breathless attention, around those orators of world-wide fame. For our own senate halls echo, and our own pulpits and hill-sides reverberate as fine specimens of elocution as ever broke its ravishing music upon the ear of mortals. And where is the human soul, clothed in fleshy habiliments, that has not, at some time, felt its mighty influence, and bowed before it as the stubborn oak bows before the gale. To master this elegant art the young man has labored in the cloistered-hall, and in the open field—has declaimed before his school-fellows and his college professor—and where one is successful, a thousand are disappointed and chagrined. Volumes have been written upon the subject—professorships have been established to teach it, and thousands of dollars expended to gain it: and disappointment has so often followed, that many have come to the *unfortunate* conclusion, that it is an endowment, or attribute of genius alone, and altogether unattainable to those who can boast of no extraordinary qualifications, or almost supernatural endowments.

But this, we take occasion to say, is a great mistake, and any individual, with *good natural powers*, (and by this we mean nothing more nor less than a healthy and normal condition of the organs concerned,) and ordinary capabilities, can cultivate it successfully. Nor are we alone in maintaining this opinion, for many successful teachers avouch the same thing. But as we design to be brief on this part of the subject, we will be content in citing the language of an able and interesting writer upon this point. Says Prof. Caldwell, "distinctness of articulation, and a clear and perfect enunciation, can rarely be said to depend upon nature at all, so entirely are they the result of education." And yet every one must perceive, that in proportion as there are physical obstacles in the way, just in that proportion, will it become a difficult acquirement, and the chances of success be proportionably diminished. But Prof. Caldwell evidently refers to those cases where all the parts concerned are simply normal, and is contending against the notion, that some *extraordinary* qualification is necessary, to cultivate, successfully, this elegant accomplishment. That the peculiar condition of the mouth and dental organs, in many individuals is a serious obstacle in the acquisition of this art, and in others, quite insurmountable, is a position which we now assume, and shall attempt to prove.

The experience and observation of every thinking man may be called to our aid in support of this position. For it cannot have escaped them, that many individuals of profound intellects and brilliant parts, make but a sorry figure in their fruitless attempts at oratory and elocution. Every one who has had experience in regard to matters of this kind, must have been conscious of great disappointment, in not realising his expectations in regard to certain distinguished men with whose writings he has long been familiar. Having fancied to himself, that because they could wield a pen so successfully, they must, therefore, be accomplished speakers, and finding himself sadly mistaken, is at a loss to account for a circumstance so strange, and apparently contradictory. But where lies the difficulty? Certainly not on the score of intellect, for their acquirements are demonstrable from their writings, which evince great strength—rigid discipline—extensive scope, and brilliancy of thought. Nor is it because they have never enjoyed the advantages of tuition, for they are fresh from the schools where elocution is taught. What, then, is the obstacle? We answer—it is to be found, in the peculiar conformation of the mouth, and the wretched condition of the teeth, giving rise to impediments and difficulties, which constitute their misfortune, and of which they are most painfully conscious.

Here, for instance, is one, whose palatine arch is greatly contracted, forming, nearly, an acute angle in front, and projecting in an unsightly manner, far beyond the circle of the inferior maxillary. The superior front teeth stand outward, while the inferior front teeth stand inward. And the only teeth that antagonise at all, are the first and second molares, so, that when the mouth is as firmly closed as it is possible to be, you can place your two fingers between the front incisores, and he cannot bite you, to save his life. The lips, if closed at all, are drawn up so as to resemble a lady's "work-bag," or reticule, and the whole, giving an expression of countenance, somewhat resembling the *Rodentia* class of animals, to which belong the rat and the squirrel.

And now I ask, did a man ever hear perfect language, from such a mouth? It matters not what his abilities may be, or his other advanges have been. His intellect may be as brilliant as that of a seraph, and yet his language will fall in murdered notes, and sounds come up from the *larynx*, in deep-toned gutturals, that never fail to fall harshly and disagreeably upon the ear of every listener.

Give him every advantage of education—place him under the tuition of the greatest elocutionist of the age, and what can he do? Place him behind the bar as a barrister, or in the pulpit as a preacher, and he still finds an insuperable barrier to the accomplishment of his purpose. He may be the cherished object of his parent's ambition—the individual, for whose education, a fortune has been expended—the object of interest to a large circle of friends, and yet it will avail him nothing, so long as he withholds from the dental surgeon the fee, which is so obviously his due. And his unsightly mouth, will stand out to the world as a monument of this indebtedness, wherever he goes.

It may be thought, by some reader of the Journal, that we have taken an extreme case. Perhaps we have. Yet we contend, and shall endeavor to show, that *every* departure from a

*perfect* denture or the most complete development of the parts concerned, is, *insomuch*, an obstacle in the acquirement of perfect elocution. But in order to a more comprehensive view of this subject, it becomes necessary to take a more minute observation of the structure and relative situation of the vocal organs, and the parts adjacent to them. This then we shall endeavor to do.

At the root of the tongue, lies a minute, semi-lunar shaped bone, which, from its resemblance to the Greek letter *υ*, or upsilon, is called the hyoid, or u-like bone. Immediately from this bone, arises a long cartilaginous tube, which extends to the lungs, and conveys the air backwards, and forwards, in the process of respiration. This tube, is the *trachea*. The upper part of it, or that immediately connected with the hyoid bone, is the *larynx*. This is formed of five distinct cartilages—the largest, and apparently, though not really, the lowermost of which produces that acute projection, or knot in the neck of males, usually called "Adams-apple." This is not a complete ring, but is open behind; the open space, being filled up, in order to make a complete ring, with two other cartilages of a smaller size and power; and which, together form the *glottis*, or aperture, and closes it in the act of swallowing so as to direct the food to the *œsophagus*, another opening, which leads to the stomach. These four cartilages are supported by a fifth, which constitutes their basis; and is narrow before, and broad behind, and has some resemblance to a seal-ring.

The *larynx* is contracted and dilated in a variety of ways by the antagonist power of different muscles, and the elasticity of its cartilaginous coats; and is covered over with a very sensible and vascular membrane, *which is a continuation of the mucous membrane of the mouth.*"*

The organ of the voice, therefore, is the larynx, its muscles, and other appendages. And the voice, itself, is the sound of the air propelled through, and striking against, the side of its glottis. And this sound is modified and varied by the least

* Vide Good's Study of Medicine.

change in the organ, or the slightest contraction of its muscles. And the shrillness or roughness of the voice, depends on the internal diameter of the glottis, its *elasticity*, *mobility*, and *lubricity;* and the force with which the air is protruded. Now, will any man contend for a moment, that the condition of the teeth and gums have nothing to do with the *elasticity*, *mobility* and *lubricity*, of the parts here under consideration? or, the diameter of the larynx itself? We think not; especially when they consider that the very *sensible* and *highly vascular* membrane which lines these organs, is but a continuation of the mucous membrane of the mouth. Nor will the intelligent dental surgeon fail to see, that the inflammation which is so often consequent on a diseased condition of the teeth, and their ulcerated fangs, may easily, nay, often does extend itself, not only along the maxillary arch, but to the fauces, the velum, the tonsil glands, and larynx, kindling up a violent disease in its track, coating the tongue with a heavy fur, and rendering every effort to speak painful, and difficult. Under such circumstances, the voice is husky, and unpleasant. The vessels of the mucous membrane are gorged with blood, the muscles, upon whose movement so much depends, become rigid, and inflexible; and the salivary glands, discharge an unhealthy secretion, which, instead of lubricating the parts, serve but to clog, and impede their action. And we say, just in proportion, to the violence of this diseased action, will be the difficulty to which we allude. As a general thing, the condition of the gums, depend upon the circumstances of the teeth. Although it sometimes is so, that the constitutional health of an individual, may very materially effect the condition of the gums. There are thousands of cases, where the teeth are so denuded, and worn down by caries, as to present to the eye of the observer, a continuation of inflamed surface, as far as the eye can extend about the throat and fauces; and that too, when the individuals themselves, can scarcely be made to believe that any especial care, or attention is demanded. This sub-acute inflammation, extending as it often does, to the trachea and larynx, cannot fail to modify, and in some measure, control their action, and as a necessary consequence, effect the modulations, and inflexions of the voice. And the extent of those circumstances of the mouth to which we here refer, is well known, to every practitioner of dental surgery, who has been a few years, in the exercise of his profession. Independent of this, there is another source of difficulty, originating in the loss of the dental organs. We must perceive, that from the very nature of the vocal organs, and the nature of sound, that the former are easily effected, and the latter as easily modified. And that the perfection of the human voice, depends upon the perfection of the vocal organs, while the modulations of voice, (in which elocution consists,) is dependent upon the form of the mouth, and condition of the teeth and parts, with which they are immediately connected. Now suppose that the larynx, the trachea, the lungs, diaphragm, and abdominal muscles, are all healthy and perfect, but, that the individual has a cleft palate, can *he* exercise his vocal organs successfully without artificial assistance? Again, suppose that his palate is sound and healthy, but, there is a sad mal-formation of the maxillary arches, like the one before mentioned, is it not obvious to every one, that this too, must constitute a very serious impediment to the perfect articulation of sound, as used in speech or language?

But even allowing, that the parts are well formed, and well developed, and that the only difficulty consists in the loss of one or more teeth, even this will present a difficulty to the easy, graceful and perfect enunciation of language, by no means trifling, or insignificant.

A circumstance, illustrative of this point occurred a few years since, in our own practice. The Rev. Mr. S. was deeply afflicted with a diseased bicuspid, situated on the right side of the superior maxillary. He called at our office for relief, we advised its extraction, and it was accordingly removed. On the following Sabbath, while engaged in the performance of divine service, he became so much annoyed by the loss of that tooth, and so difficult was his enunciation, that he was compelled to stop, in the midst of his discourse, and explain the cause of his difficulty, to his congregation. And this the loss of a single tooth. But what must be the inconvenience, where there is scarcely a perfect tooth remaining, where ragged edges of decayed teeth, fret, and lacerate the tongue, and where dead roots, and ulcerated fangs, render the parts inflamed, and exceedingly irritable. In such cases, the air comes rushing through the trachea into the larynx, through the larynx into the fauces, it is there held in abeyance by the tongue and muscular coats of the mouth, until it can be modulated into proper sounds; but the tongue, in its effort to do this, encounters difficulties hard to appreciate, and stubborn to subdue. Overleaping its natural province, having no teeth to restrain and circumscribe its movements, it murders the very sounds it would fain control, and gives birth to notes, the most discordant and unmusical. Nature's own beautiful barricade is broken down, and the voice comes hissing through the crevices, and dilapidated walls, somewhat as the storm-spirit howls through the dilapidated apartments of an old building. How different all this, from that rich sonorous language, that falls so gracefully, in measured notes, from the lips of one whose broad expanded mouth and well developed teeth, enables him to mould his voice, in sounds the most mellifluous and delightful. No squeaking of the voice, no hissing of the air, no unnatural contortion of the muscles of the mouth to accommodate it to its unnatural circumstances; giving rise to habits that are as painful to the individual, as they are unpleasant and disagreeable to others. Such a mouth has Henry Clay of Kentucky, such a mouth had Patrick Henry, and such a mouth had Daniel O'Connell. Indeed, it is quite impossible to call to mind a single renowned orator who does not in the main, possess these physical characteristics. Who ever heard of *one* with a small, contracted mouth, shaped like that of the rat or squirrel? Either such a mouth or face, are seldom associated with great thoughts, much less with elegant language. Speaking of Henry Clay, we well remember, that his *mouth* has been the subject of frequent remark; and many amusing anecdotes, of his peculiar expression of countenance, connected with the expansion of his mouth, when engaged in debate, have been related of him.

And there can be no question, but that he is much indebted to his mouth, and the peculiar play of his features, for his great success in oratory. The lingering playful smile, the biting sarcasm—the expression of supreme contempt—the supercilious curl of the lip—the silver accents of his voice—the delightful enunciation—the elegant diction—all, all, are much more than appears to a superficial observer, dependent on a perfect denture. The loss of the front inferior incisores, more especially, gives rise to difficulty, in the articulation of language. To be satisfied of this, says Dr. Robinson, (see Robinson on the Teeth, p. 163,) you have only to pronounce, distinctly, some such words as the following, "*this*," "*therefore*," "*they*," "*those*," or "*that*," and you will immediately perceive the vibratory action of the tongue, on these teeth. The absence of these teeth, it must be perceived, would allow the air to escape, and thus produce a lisping sound that greatly mars the beauty, and force of language. That the force and strength of habit, may overcome many difficulties I am prepared to admit, and that the mouth will accommodate itself to circumstances in the formation of sounds, to a certain extent, I will also allow, but in doing this, it often gives rise to other evils of equal, if not greater magnitude. In a public speaker, disagreeable habits greatly detract from his effectiveness. Various contortions of the mouth and face, produced by the effort to accommodate itself to the loss of the teeth, often occasions disagreeable sensations in the hearer, and results, in a loss of sympathy between the speaker and his hearers, which sympathy is very necessary, to render public speaking effective, and useful. So comical and ludicrous, are these expressions of the countenance at times, that

"To be grave, exceeds all power of face."

To see a man shape his lips, to fit a word, and struggle to expel it, as if he had something disagreeable in his mouth, and was about to strangle under its influence, is always painful, and laborious. And we are often reminded of a story, told by a facetious and witty friend, of a certain shrewd Yankee, who, finding himself in the western country, in rather destitute circumstances, hit upon an expedient to "raise the wind," by teaching a *whistling* school. Having assembled his pupils, and arranged them in regular order, he told them, the first thing was, to "prepare to pucker."—So it is very frequently, with this class of speakers, the peculiar circumstances of their mouths, require of them, that they "prepare to pucker."—And hence it is, that you will often see them, with their lips compressed nearly two-thirds their length, and opening in one corner, like the mouth of a trumpet, expelling their words through that opening, in the most laborious manner. And these habits, which are so easily contracted, generally remain through life. In almost all cases, they are referable, either to a crowded condition of the teeth, and consequent irregularity, or to a loss of one or more of these important organs.

There is another consideration, which gives additional importance to the dental organs, in connection with the subject of elocution. It is considered at some length in Dr. Robinson's very excellent work on the teeth, above referred to; and to that work we refer the reader, who may be curious to see the Doctor's reasoning upon that subject. It is the circumstance of the teeth's being in a modified sense, organs of hearing, or rather, conductors of sound; giving to the individual, in some degree, an appreciation of the sound of his own voice, which is so essential to its modulation.

What we have said in relation to the teeth, as being so important in the exercise of articulate language, or speech, is equally true of the vocal organs, when employed in singing, and of the teeth, in respect to song. The same difficulties originate in the one case, as in the other. The voice is modulated, and all its delicate inflexions in a great measure controlled by the circumstances of which we have been speaking, and we know not that it is necessary to consume any more time, in attempting to illustrate this part of our subject. Suffice it to say, that with the same difficulties existing in regard to the teeth, gums, and buccal cavity as above referred to, our efforts at song, will be equally unsuccessful, and abortive, and productive of the same unpleasant habits, and disagreeable contortions.

And here, if we mistake not, we have the source and origin of those disagreeable habits, into which so many thousands of our race have fallen, and which have been so productive of evil to a very large number of clergymen, and others engaged in public speaking. Resulting in diseases of the throat, of a most painful and afflicting character, and not unfrequently, the entire use of the voice, has been lost, from such diseases of the vocal organs. *Bronchitis*, *laryngitis* and *phthisis-pulmonalis*, are the scourge of public speakers of the present day, and in our humble judgment, they are almost uniformly the result of circumstances to which we have alluded. The premature decay, and loss of the teeth, and the diseases of the mouth and throat resulting therefrom, and those malformations of the mouth and jaws, which are mainly the result of neglect, or mismanagement during the process of dentition, must, of necessity, make the use of language, hard and laborious. The parts are absolutely twisted out of their natural relations, and every use of them under such circumstances becomes a strain, which is sure to result in disease. For the necessary sounds cannot be formed, unless the parts are accommodated, and the effort to do this, brings them into a relation to each other, which is forced and unnatural. The muscles of the throat and larynx, are overtaxed in the performance of their office, and not being able continually to maintain the necessary degree of tension, they become relaxed, flaccid, and inflamed. And, as a natural consequence, the voice becomes husky and unpleasant, and of course, every succeeding effort only aggravates the difficulty. The irritation thus produced about the larynx and trachea, induces a cough, and the afflicted patient runs down with consumption. And thus it is, that thousands of our most useful men are cut off, in the very prime of life, and the zenith of their bright career.

And now, if the view which we have taken of this subject be correct, (and whether correct or not, we are deeply impressed with its importance,) it must certainly furnish some considerations, which are worthy the attention, not only of the dental surgeon, but of the physiologist and medical practitioner, who are so frequently called upon to treat such cases as these.

From all we have written upon this subject, we think it must be obvious to every intelligent reader, that the duties of the dental surgeon, are neither mean, or unimportant, as he must necessarily hold, in the exercise of his profession, the absolute destiny of many of his race. The germ of future greatness, in his infant patient, may be entirely crushed, or developed under his hands, and the hope, and expectation of parental ambition and fondness blasted forever, or fully realized. The pulpit and the altar—the bar and the forum, are alike dependent upon him, for their highest, and brightest ornaments, and if he will, he may cheat the sexton and the grave-yard of many a victim.

Again, in view of this subject, how reprehensible is the conduct of many parents, who, while they are lavishing thousands of dollars upon their children, in order to give them a "*fashionable*" education, at the same time withhold, or grudgingly bestow, the insignificant fee required by the dentist. And who, if they employ him at all, are sure to seek out one, who will do his work the "*cheapest*," irrespective of his qualifications. Bestowing thousands where it is not needed, nay, worse than that, where it is directly hurtful, and withholding it, where it is most essential. Such parents are unworthy to have the care of children, for they literally, "know not what they do."

That our useful art possesses resources that are not yet developed, especially to the extent of which they are capable, is perfectly obvious. We trust the time is not far distant, when these resources will be acknowledged and appreciated as they deserve to be. Who questions the propriety of a teacher of elocution in our colleges and public schools? And yet, *who* supposes that an accomplished dental surgeon is *much more* necessary. And without whose services, the professor of elocution may labor till "dooms-day," with very little result, which is in any degree satisfactory. Also, in our numerous female seminaries, and common schools, where music becomes an important part of education, the same thing is eqally true, equally important; more so, indeed, for

"Just as the twig is bent, the tree is inclined."

And as there is no period of life, in which the services of the dental surgeon is so necessary, as in youth, so there is no period, when they are so fruitful of good to the patient.

